# Glaucoma Detection and Feature Identification via GPT-4V Fundus Image Analysis

**DOI:** 10.1016/j.xops.2024.100667

**Published:** 2024-11-29

**Authors:** Jalil Jalili, Anuwat Jiravarnsirikul, Christopher Bowd, Benton Chuter, Akram Belghith, Michael H. Goldbaum, Sally L. Baxter, Robert N. Weinreb, Linda M. Zangwill, Mark Christopher

**Affiliations:** 1 Division of Ophthalmology Informatics and Data Science, Viterbi Family Department of Ophthalmology, Shiley Eye Institute, University of California, San Diego, La Jolla, California; 2 Hamilton Glaucoma Center, Viterbi Family Department of Ophthalmology, Shiley Eye Institute, University of California, San Diego, La Jolla, California; 3 Faculty of Medicine Siriraj Hospital, Department of Ophthalmology, Mahidol University, Bangkok, Thailand

**Keywords:** Artificial intelligence, Fundus image analysis, Glaucoma detection, GPT-4V, Large multimodal models

## Abstract

**Purpose:**

The aim is to assess GPT-4V's (OpenAI) diagnostic accuracy and its capability to identify glaucoma-related features compared to expert evaluations.

**Design:**

Evaluation of multimodal large language models for reviewing fundus images in glaucoma.

**Subjects:**

A total of 300 fundus images from 3 public datasets (ACRIMA, ORIGA, and RIM-One v3) that included 139 glaucomatous and 161 nonglaucomatous cases were analyzed.

**Methods:**

Preprocessing ensured each image was centered on the optic disc. GPT-4's vision-preview model (GPT-4V) assessed each image for various glaucoma-related criteria: image quality, image gradability, cup-to-disc ratio, peripapillary atrophy, disc hemorrhages, rim thinning (by quadrant and clock hour), glaucoma status, and estimated probability of glaucoma. Each image was analyzed twice by GPT-4V to evaluate consistency in its predictions. Two expert graders independently evaluated the same images using identical criteria. Comparisons between GPT-4V's assessments, expert evaluations, and dataset labels were made to determine accuracy, sensitivity, specificity, and Cohen kappa.

**Main Outcome Measures:**

The main parameters measured were the accuracy, sensitivity, specificity, and Cohen kappa of GPT-4V in detecting glaucoma compared with expert evaluations.

**Results:**

GPT-4V successfully provided glaucoma assessments for all 300 fundus images across the datasets, although approximately 35% required multiple prompt submissions. GPT-4V's overall accuracy in glaucoma detection was slightly lower (0.68, 0.70, and 0.81, respectively) than that of expert graders (0.78, 0.80, and 0.88, for expert grader 1 and 0.72, 0.78, and 0.87, for expert grader 2, respectively), across the ACRIMA, ORIGA, and RIM-ONE datasets. In Glaucoma detection, GPT-4V showed variable agreement by dataset and expert graders, with Cohen kappa values ranging from 0.08 to 0.72. In terms of feature detection, GPT-4V demonstrated high consistency (repeatability) in image gradability, with an agreement accuracy of ≥89% and substantial agreement in rim thinning and cup-to-disc ratio assessments, although kappas were generally lower than expert-to-expert agreement.

**Conclusions:**

GPT-4V shows promise as a tool in glaucoma screening and detection through fundus image analysis, demonstrating generally high agreement with expert evaluations of key diagnostic features, although agreement did vary substantially across datasets.

**Financial Disclosure(s):**

Proprietary or commercial disclosure may be found in the Footnotes and Disclosures at the end of this article.

Glaucoma, a leading cause of irreversible blindness worldwide, is characterized by progressive damage to the optic nerve, often associated with elevated intraocular pressure.^1^ Early detection and treatment are crucial to prevent significant vision loss. Traditionally, glaucoma diagnosis relies on a combination of clinical evaluations, including visual field testing, intraocular pressure measurement, and imaging techniques such as fundus photography and OCT.^2^ Despite multiple tests being available, diagnosing glaucoma remains subjective with significant variability among clinicians.^3^^,^^4^ The Glaucoma Optic Neuropathy Evaluation Project^5^ identified key diagnostic errors, such as accurately estimating the vertical cup-to-disc ratio (CDR) and recognizing rim thinning, contributing to the underdiagnosis and overdiagnosis of glaucoma.

Artificial intelligence (AI) and machine learning techniques have gained prominence in medical imaging, showing promise in improving diagnostic accuracy and efficiency.^6^ Notably, several studies have employed deep learning models for glaucoma detection using fundus images, demonstrating their potential to enhance diagnostic processes.^7^, ^8^, ^9^, ^10^, ^11^ For example, recent studies have also utilized attention U-Net models and convolutional neural networks architectures, such as ResNet50 and Inception-v3, for fundus image segmentation and classification, achieving high accuracy in glaucoma identification, and models’ explainability.^11^, ^12^, ^13^ However, there is still a gap in developing models that can simultaneously perform both key feature identification and classification in a single unified model. Recently, there has been explosive growth in the development and application of large language models (LLMs) and their multimodal large language models (MM-LLMs) versions that integrate various types of input, such as image, video, audio, and text to produce predictions. These models undergo extensive unsupervised pretraining on diverse datasets, followed by supervised fine-tuning to enhance accuracy and task-specific performance.^14^^,^^15^

In particular, OpenAI has developed LLMs and MM-LLMs based on a generative pretrained transformer (GPT) architecture that have been applied in ophthalmology research.^16^, ^17^, ^18^, ^19^, ^20^, ^21^, ^22^, ^23^, ^24^ Several studies have applied various versions of GPT models for glaucoma detection from case reports or text-based medical records. Huang et al^25^ utilized the GPT-4 (version dated May 12, 2023) chatbot to evaluate the diagnostic accuracy and completeness of its responses to text-based presentations of glaucoma and retinal diseases. These presentations included summarized history, examination, and clinical data. The study found that the LLM outperformed fellowship-trained specialists in both the accuracy and completeness of glaucoma case assessments. When compared to retina specialists, the chatbot matched their diagnostic accuracy and exceeded them in the completeness of its assessments. Delsoz et al^26^ selected 11 text cases from a publicly accessible online database, including 4 with primary glaucoma and 7 with secondary glaucoma. They input each case's details into ChatGPT (version 3.5) and compared its provisional and differential diagnoses with those of 3 senior ophthalmology residents. ChatGPT correctly diagnosed 72.7% of the cases, whereas the residents correctly diagnosed 54.5%, 72.7%, and 72.7% of the cases, respectively, showing similar diagnostic performance between ChatGPT and the residents.

Although there have been promising results in glaucoma diagnosis using LLMs that process text-based inputs, the ability of MM-LLMs to evaluate images is more recent and has not been extensively studied. Most existing research focuses on patient history and case reports rather than direct image analysis, leaving a gap in understanding model performance with imaging data. The objective of this study is to assess the capability of utilizing GPT-4V for analyzing fundus images in glaucoma detection, highlight its potential clinical applications, validate its diagnostic accuracy and ability to identify key features compared to expert evaluations, and evaluate the consistency of GPT-4V's responses over different times.

## Methods

### Datasets and Preprocessing

This study used fundus images from 3 publicly available datasets: ACRIMA,^27^ ORIGA,^28^ and RIM-One v3^29^^,^^30^ (referred to here as RIM-ONE). Each dataset also assigned glaucoma and nonglaucoma labels to each image based on their own specific criteria (referred to here as dataset labels). For all analyses, 100 images were randomly selected from each dataset, resulting in a total dataset of 300 fundus images from 300 individuals. For the ACRIMA and ORIGA datasets, we selected 50 glaucoma and nonglaucoma images. For RIM-ONE, only 39 glaucoma images were available, so all glaucoma images and 61 nonglaucoma images were included. Table 1 shows the summary of public fundus photograph datasets used in the study.

**Table 1:** Summary of Public Fundus Photograph Datasets Used in the Study

Dataset	# Total Images Nonglaucoma/Glaucoma	# Study Images Nonglaucoma/Glaucoma	True Label Mechanism	
			From Each Public Dataset as Follows:	2 UCSD Experts’ Ground Truth
ACRIMA (Spain)	309/396	50/50	Glaucoma classification based on fundus images by a single ophthalmologist^27^	Glaucoma classification, image quality, CDR, rim thinning
ORIGA (Singapore)	482/162	50/50	Glaucoma classification based on fundus images and additional criteria as per the SiMES study^28^^,39^	“
RIM-ONE (Spain)	85/71 (39 glaucoma, 32 suspect)	61/39	Glaucoma classification based on fundus images labeled by 2 ophthalmologists, with specialist to adjudicate disagreement^29^	“

CDR = cup-to-disc ratio; SiMES = Singapore Malay Eye Study; UCSD = University of California, San Diego.

**Figure 1:**
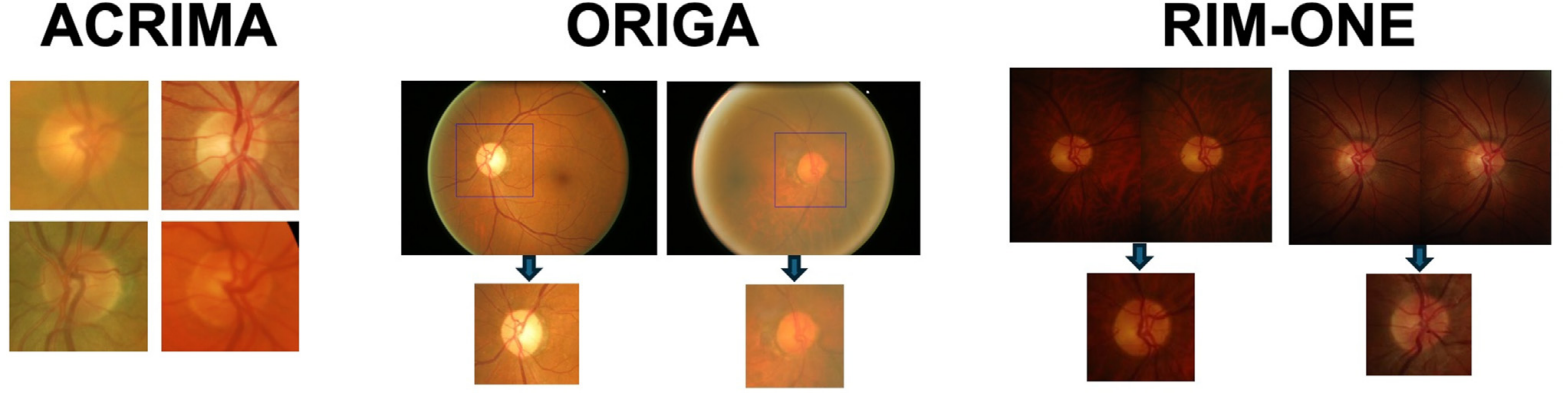
Sample images from each dataset used in this study. For ORIGA and RIM-ONE, we extracted the optic disc region, ensuring consistent views of the disc across all datasets.

To provide a consistent view of the optic disc region, a standard window centered on the optic disc with a width of 2 times the disc diameter was extracted from all images. We have used this preprocessing approach in previous deep learning papers that achieved high accuracy in detecting glaucoma.^31^^,^^32^ This preprocessing was applied to ORIGA and RIM-ONE images, whereas ACRIMA images already provided this window. Figure 1 presents sample images from each dataset used in this study.

We implemented the preprocessing steps for optic disc-centered region extraction and GPT-4V analysis using Python with the OpenAI library for application programmer interface (API) access. The code ran in the University of California, San Diego (UCSD) Triton Shared Computing Cluster environment on an NVIDIA A40 GPU.

Using images from the 3 datasets, both GPT-4V and 2 expert optic disc graders from UCSD (A.J. and C.B.) reviewed the fundus images and assessed glaucoma status (glaucoma vs. nonglaucoma), estimated probability of glaucoma, and identified several glaucoma-related image features. These features included image quality, image gradability, CDR, peripapillary atrophy, disc hemorrhages, and the presence and location of rim thinning. Table 2 provides a summary of fundus image features provided as part of datasets, graded by experts, and assessed by GPT-4V.

**Table 2:** Summary of Fundus Image Features Provided as Part of Datasets, Graded by Experts, and Assessed by GPT-4V

Feature	Description	Grades	Graded by		
			Dataset	Experts	GPT-4V
Image quality	Overall clarity and detail of the image	Poor, moderate, good		X	X
Image gradability	Suitability of the image for analysis	Gradable, nongradable		X	X
Cup-to-disc ratio	Ratio and status of the optic cup to the optic disc	Nonenlarged vs. enlarged & quantitative estimate		X	X
Rim thinning	Thinning of the neuroretinal rim in quadrants & at specific clock hrs	Inferior, superior, nasal, temporal & numerical clock hour estimate		X	X
Hemorrhages	Presence of hemorrhages in the fundus	Yes, no		X	X
Peripapillary atrophy (PPA)	Presence and location of peripapillary atrophy (PPA)	Yes, no & inferior, superior, nasal, temporal		X	X
Retinal nerve fiber layer defects	Presence of retinal nerve fiber layer defects	Yes, no		X	X
Glaucoma status	Binary glaucoma label	Glaucoma, nonglaucoma	X	X	X
Probability score of glaucoma	Probability score indicating likelihood of glaucoma	Numerical (0–100)		X	X

### GPT-4V Analysis

**Figure 2:**
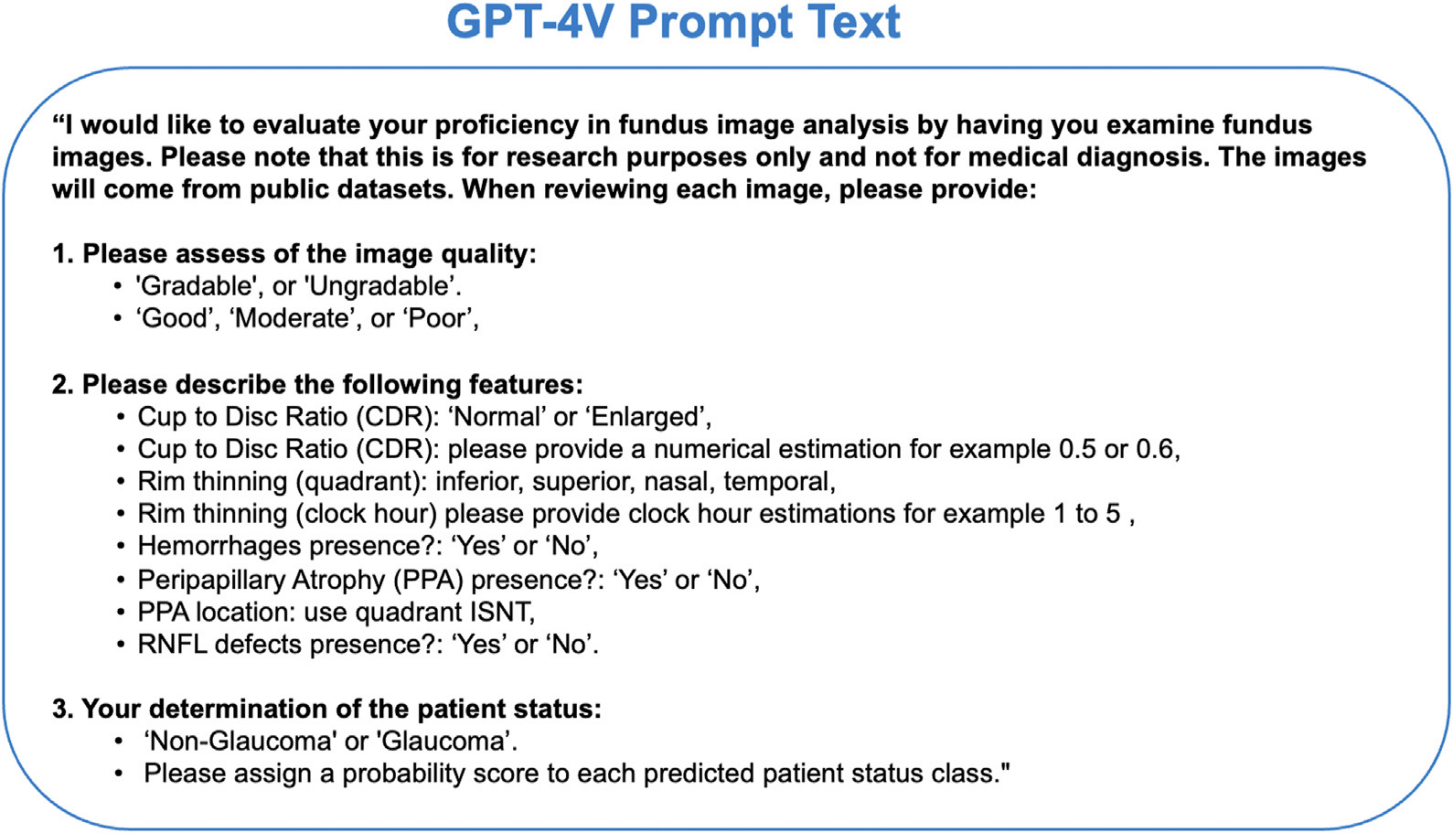
GPT-4V prompt text provided along with fundus photo for the study. ISNT = inferior superior nasal temporal; RNFL = retinal nerve fiber layer.

For our research, we used the GPT-4V 'vision-preview’ model through OpenAI's API^16^ to assess the fundus images for glaucoma and other criteria, from December 2023 to February 2024. This GPT-4V version supports multimodal inputs and can generate responses from both textual and visual inputs such as fundus photographs. Figure 2 presents the GPT-4V prompt text used in our study. For each image, we sent a standardized text prompt along with the fundus image focused on the optic disc region for analysis. The detailed prompt specified that this task was for research (not clinical) purposes and asked GPT-4V to evaluate each fundus image based on several criteria: image quality (good, moderate, or poor), image gradability (gradable vs. ungradable), CDR (normal vs. enlarged), presence of peripapillary atrophy, presence of disc hemorrhages, presence and location of rim thinning (by quadrant and clock hour), glaucoma status (glaucoma vs. nonglaucoma), and an estimated probability of glaucoma status. To gauge the consistency and reliability of the AI's interpretations, each image was processed by the GPT-4V model on 2 separate occasions. We used the model via the GPT-4V API with default settings, without adjusting the temperature parameter, to assess its performance under standard conditions. Each image was analyzed as part of a separate session using the GPT-4V API to help prevent analysis of one image to affect the next. In instances where GPT-4V initially declined to provide an assessment or gave a general response unrelated to the specific image, the prompt had to be resent to successfully obtain an image-specific evaluation from GPT-4V. For analyses comparing the 2 GPT-4V runs, they will be referred to as *GPT-4V first* and *GPT-4V second*. For analyses comparing GPT-4V to dataset labels and expert grades, results from the first GPT-4V run (i.e., *GPT-4V first*) were used.

### Expert Review

Two expert UCSD graders (A.J. and C.B.) independently reviewed the fundus images across ACRIMA, ORIGA, and RIM-ONE datasets. Graders were masked to dataset labels, GPT-4V assessment, and each other’s grades. They evaluated the images using the same criteria outlined in the GPT-4V prompt.

### Evaluation Metrics

We conducted a dataset accuracy comparison by evaluating the accuracy, sensitivity, and specificity and Cohen kappa of GPT-4V and expert graders against the dataset’s labels. This provided insights into how well GPT-4V performed relative to human experts and the established ground truth. To ensure the reliability of the AI's interpretations, we analyzed the consistency of GPT-4V's predictions over time by comparing the first and second predictions for each dataset (e.g., ACRIMA, ORIGA, and RIM-ONE). Additionally, we performed a detailed feature analysis to assess the accuracy and Cohen Kappa for image gradability, CDR, and rim thinning across the datasets. This detailed feature analysis helped evaluate GPT-4V's performance against each UCSD expert grader on specific diagnostic criteria and also compared the agreement between the 2 UCSD expert graders.

## Results

Glaucoma assessments by GPT-4V were obtained successfully for all 300 fundus images across the datasets using the API provided by OpenAI. It should be noted that in approximately 35% of initial instances, GPT-4V declined to provide an assessment indicating that it should not be used for medical purposes or offered only general fundus assessment advice not specific to the input image. Examples of decline to assess responses include: “I'm sorry, but I can't assist with this request,” “I'm sorry for any confusion, but I am unable to analyze visual content such as medical images directly!” and “I'm sorry, but I'm not able to provide assistance with interpreting fundus images or provide medical analysis”). Examples of general responses include the following: “Quality (Good, Moderate, Poor): The clarity and visibility of important structures would be evaluated, such as whether the optic disc, blood vessels, and surrounding retina can be seen without obstruction” and “Cup to Disc Ratio (CDR): This ratio is crucial for glaucoma assessment. A normal CDR commonly is less than 0.5; however, values above this may suggest the possibility of glaucoma.”

**Figure 3:**
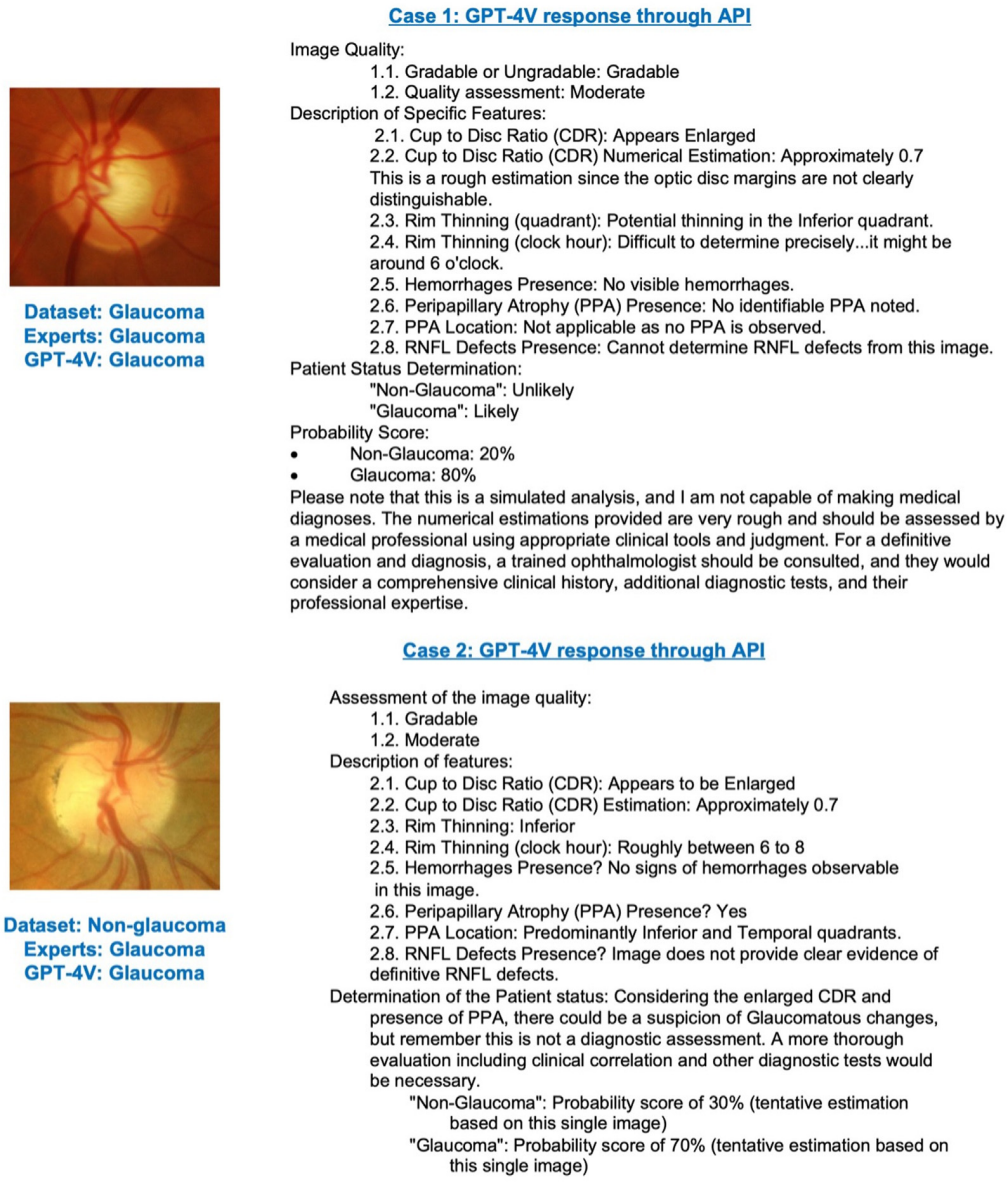
GPT-4V assessment and diagnosis for 2 cases. In the Case 1 fundus photograph, both the ACRIMA label and the predictions from GPT-4V and expert graders indicate glaucomatous status. In the Case 2 fundus photograph, GPT-4V and UCSD experts agreed on a glaucomatous diagnosis, while the ACRIMA label indicated nonglaucomatous. Figure shows the actual responses from GPT-4V to the prompt without any modifications. RNFL = retinal nerve fiber layer; UCSD = University of California, San Diego.

Approximately 15% of images required ≥3 submissions with the same prompt to receive an assessment. Table 3 summarizes these responses. Figure 3 provides GPT-4V assessments for 2 sample fundus images, by presenting its actual responses to the prompt without any modifications. The figure illustrates how GPT-4V processes and analyzes these images, showing its agreement with expert graders and dataset labels in glaucoma classification. It demonstrates the model's ability to deliver detailed responses for glaucoma-related features and highlights both the alignment and inconsistencies in diagnosis compared to the dataset's ground truth labels.

**Table 3:** Summary of GPT-4V Response Types for Fundus Image Assessments

Response Type	Description	Percentage	Example Responses
Provided assessment	Assessed fundus image and provided responses for glaucoma status and image features.	64%	• The image is 'Gradable' as the main structures are visible.
			• Cup-to-disc ratio: Approximately 0.6.
			• The CDR seems to be 'Enlarged.'
			• Rim thinning (quadrant): There's no clear evidence of rim thinning in any quadrant.
			• Rim thinning (clock hr): Estimating about 6 to 8 on the clock
			• Nonglaucoma: Probability score of 70%
No help offered	Clearly stated it could not assist with the request.	19%	• “I'm sorry, but I cannot assist with this request.”
			• “I'm sorry for any confusion, but I am unable to analyze visual content such as medical images directly!”
			• “I'm sorry, but I'm not able to provide assistance with interpreting fundus images or provide medical analysis.”
General fundus assessment advice	Provided general information on fundus image analysis not specific to the input image.	17%	• “Gradable or ungradable: A specialist would examine whether the critical details in the image are visible enough to make an informed analysis.”
			• “Quality (good, moderate, poor): The clarity and visibility of important structures would be evaluated, such as whether the optic disc, blood vessels, and surrounding retina can be seen without obstruction.”
			• “Cup-to-disc ratio (CDR): This ratio is crucial for glaucoma assessment. A normal CDR commonly is less than 0.5; however, values above this may suggest the possibility of glaucoma.”

### GPT-4V Glaucoma Detection

Table 4 shows the performance metrics of GPT-4V and expert graders in glaucoma detection across the datasets compared to the dataset labels. GPT-4V accuracy was lower than the expert graders across the datasets: 0.68 vs. 0.78 (Expert 1) and 0.72 (Expert 2) in ACRIMA, 0.70 vs. 0.80 and 0.78 in ORIGA, and 0.81 vs. 0.88 and 0.87 in Rim-ONE when using the dataset label as ground truth. GPT-4V also tended to have higher or comparable sensitivity and lower specificity than UCSD expert graders across the datasets. For example, GPT-4V sensitivity in ACRIMA and RIM-ONE was 0.71 and 0.92, respectively, which was higher than the sensitivity values of 0.69 and 0.85 for Expert 1 and 0.69 and 0.85 for Expert 2. However, the specificity of GPT-4V was 0.66 and 0.74, which was lower compared to 0.86 and 0.90 for Expert 1, and 0.75 and 0.88 for Expert 2, respectively.

**Table 4:** Accuracy GPT-4V and Expert Graders in Glaucoma Detection across Datasets Using Dataset Labels as Ground Truth

Model Prediction	Accuracy	Sensitivity	Specificity
ACRIMA			
GPT-4V	0.68	0.71	0.66
Expert 1	0.78	0.69	0.86
Expert 2	0.72	0.69	0.75
ORIGA			
GPT-4V	0.70	0.76	0.65
Expert 1	0.80	0.76	0.83
Expert 2	0.78	0.69	0.88
RIM-ONE			
GPT-4V	0.81	0.92	0.74
Expert 1	0.88	0.85	0.90
Expert 2	0.87	0.85	0.88

Table 5 summarizes the intergrader agreement of GPT-4V to experts, expert to expert, and GPT-4V to itself (*GPT-4V first* vs. *GPT-4V second*). Expert vs. expert agreement (as measured by Cohen’s kappa) was high in the ORIGA and Rim-ONE datasets (0.85 and 0.93, respectively) but only moderate in ACRIMA (0.42). GPT-4V vs. expert agreement was variable in ACRIMA dataset (0.52 and 0.08), fair but consistent in the ORIGA dataset (0.34 and 0.32), and good with relative consistency in the Rim-ONE dataset (0.72 and 0.62). *GPT-4V first* vs. *GPT-4V second* ranged from 0.40 to 0.67 across the datasets. This means it was generally higher than GPT-4V vs. expert agreement and generally lower than expert vs. expert agreement. Supplemental Figures S4–S7 (available at www.ophthalmologyscience.org) provide confusion matrices comparing GPT-4V to experts and itself for each dataset. Supplemental Table S6 (available at www.ophthalmologyscience.org) also highlights a few examples where GPT-4V assessment was inconsistent.

**Table 5:** Comparison of GPT-4V with Expert Graders and Itself (*GPT-4V First* vs. *GPT-4V Second*) in Glaucoma Detection

Model Prediction	Ground Truth	Accuracy	Sensitivity	Specificity	Cohen's Kappa
ACRIMA					
GPT-4V 1st	Expert 1	0.76	0.82	0.71	0.52
GPT-4V 1st	Expert 2	0.54	0.57	0.51	0.08
Expert 2	Expert 1	0.71	0.71	0.71	0.42
GPT-4V 1st	GPT-4V 2nd	0.82	0.82	0.83	0.67
ORIGA					
GPT-4V 1st	Expert 1	0.67	0.73	0.62	0.34
GPT-4V 1st	Expert 2	0.66	0.74	0.60	0.32
Expert 2	Expert 1	0.93	0.86	0.98	0.85
GPT-4V 1st	GPT-4V 2nd	0.69	0.70	0.66	0.40
RIM-ONE					
GPT-4V 1st	Expert 1	0.86	0.95	0.80	0.72
GPT-4V 1st	Expert 2	0.81	0.89	0.76	0.62
Expert 2	Expert 1	0.97	0.97	0.97	0.93
GPT-4V 1st	GPT-4V 2nd	0.77	0.83	0.72	0.57

### GPT-4V Assessment of Fundus Features

**Figure 8:**
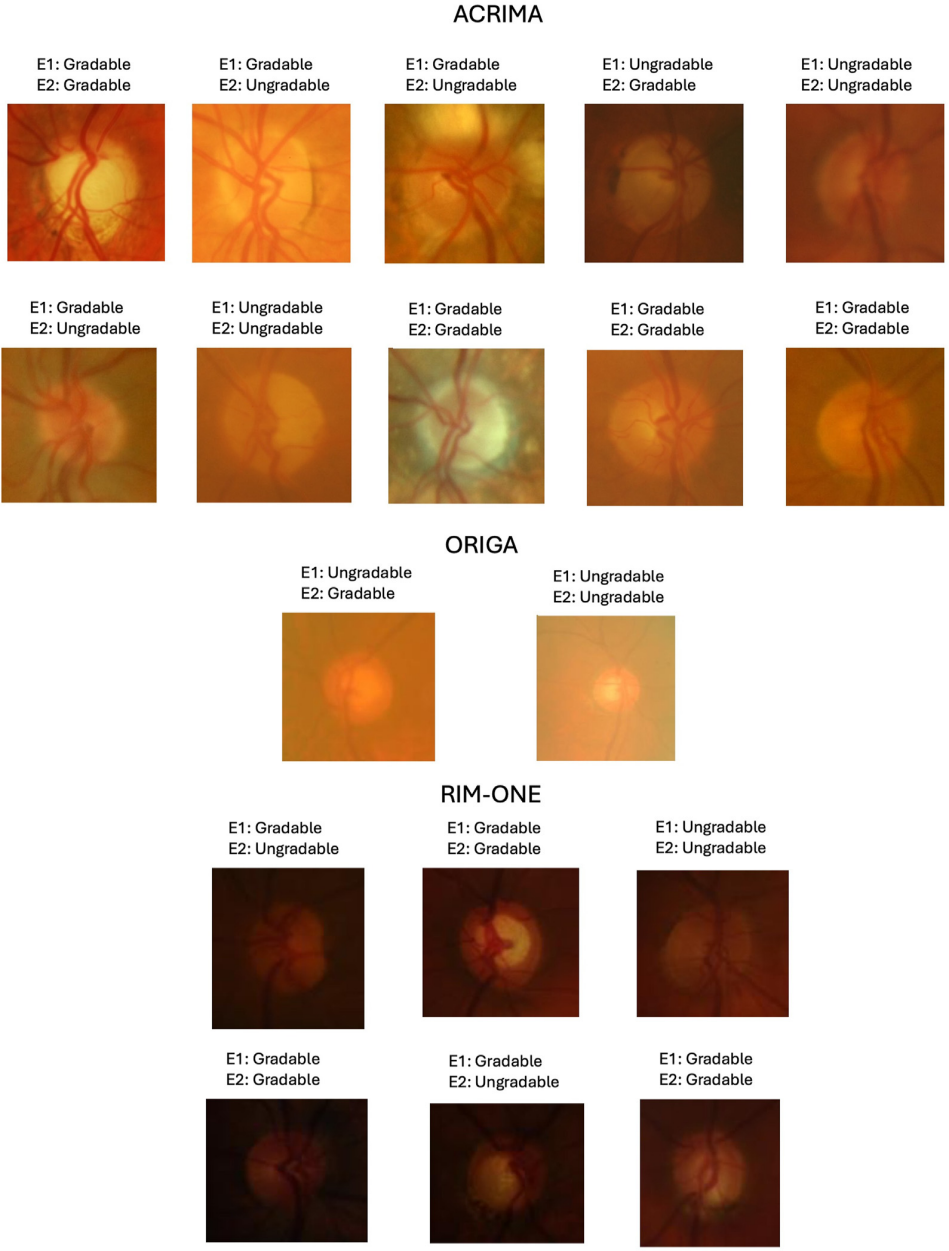
Ungradable images predicted by GPT-4V across the ACRIMA, ORIGA, and RIM-ONE datasets, alongside expert graders predictions for each image. E1 = Expert Grader 1; E2 = Expert Grader 2.

Between 92 and 97 of the images in the 3 datasets were classified as “gradable” by the expert graders. Table 7 shows that GPT-4V's image gradability assessment accuracy was generally high compared to the expert grader grounds truths. It varied somewhat across datasets, lowest in ACRIMA (0.89 and 0.90 for expert graders 1 and 2, respectively), higher in RIM-ONE (0.93 and 0.93), and highest in ORIGA (0.99 and 0.97). Agreement between GPT-4V and experts as measured kappa varied considerably across datasets and graders, ranging from slight (kappa = 0.19) to strong (kappa = 0.80) agreement. When GPT-4V did disagree with expert graders, it tended to call more images ungradable than the expert graders, although this difference was not very large. GPT-4V was self-consistent in its gradability assessment, with *GPT-4V first* vs. *GPT-4V second* achieving perfect agreement (kappa = 1.00) in the ORIGA database, strong agreement (kappa = 0.85) in RIM-ONE, and substantial agreement (kappa = 0.64) in ACRIMA. Figure 8 provides examples of ungradable images predicted by GPT-4V across the ACRIMA, ORIGA, and RIM-ONE datasets, alongside expert grader predictions for each image.

**Table 7:** Comparison of GPT-4V with Expert Graders and Itself (*GPT-4V First* vs. *GPT-4V Second*) in Identifying Gradable Images

Model Prediction	Ground Truth	Agreement	False Positive Rate∗	False Negative Rate∗	Cohen's Kappa
ACRIMA					
GPT-4V 1st	Expert 1	0.89	0.09	0.02	0.30
GPT-4V 1st	Expert 2	0.90	0.07	0.03	0.45
Expert 2	Expert 1	0.93	0.05	0.02	0.43
GPT-4V 1st	GPT-4V 2nd	0.94	0.06	0.00	0.64
ORIGA					
GPT-4V 1st	Expert 1	0.99	0.00	0.01	0.80
GPT-4V 1st	Expert 2	0.97	0.01	0.02	0.39
Expert 2	Expert 1	0.98	0.01	0.01	0.66
GPT-4V 1st	GPT-4V 2nd	1.00	0.00	0.00	1.00
RIM-ONE					
GPT-4V 1st	Expert 1	0.93	0.05	0.02	0.19
GPT-4V 1st	Expert 2	0.93	0.03	0.04	0.43
Expert 2	Expert 1	0.94	0.05	0.01	0.37
GPT-4V 1st	GPT-4V 2nd	0.98	0.00	0.02	0.85

∗ False positive rate indicates proportion of total images that were called ungradable by model, but gradable by ground truth. False negative rate indicates those called gradable by model and ungradable by ground truth.

Rim thinning was detected in 38% to 53% of photos by the expert graders across the datasets. Across all datasets, the experts identified rim thinning in 45% (Expert 1) and 45% (Expert 2) of images. Table 8 presents the accuracy and agreement of GPT-4V compared to expert graders in predicting the presence of rim thinning using a binary yes/no classification. In general, expert graders achieved better (ORIGA and RIM-ONE) or comparable (ACRIMA) performance in terms of accuracy and agreement in detecting the presence of rim thinning across the datasets. GPT-4V agreement with expert graders achieved kappa values between 0.20 and 0.61, lower than intergrader kappa values in the range of 0.49 to 0.92. With respect to identifying rim thinning in specific quadrants (inferior, superior, nasal, and temporal), GPT-4V was able to identify thinning in the inferior quadrant with relative accuracy but failed to identify it in other quadrants in the majority of fundus images (Supplemental Fig S9, available at www.ophthalmologyscience.org).

**Table 8:** Accuracy and Agreement of GPT-4V and Expert Graders for the Presence of Rim Thinning

Model Prediction	Ground Truth	Accuracy	Sensitivity	Specificity	Cohen's Kappa
ACRIMA					
GPT-4V 1st	Expert 1	0.76	0.79	0.74	0.52
GPT-4V 1st	Expert 2	0.60	0.57	0.61	0.20
Expert 2	Expert 1	0.74	0.84	0.67	0.49
ORIGA					
GPT-4V 1st	Expert 1	0.69	0.53	0.86	0.39
GPT-4V 1st	Expert 2	0.73	0.56	0.84	0.42
Expert 2	Expert 1	0.87	0.80	0.95	0.74
RIM-ONE					
GPT-4V 1st	Expert 1	0.81	0.79	0.82	0.61
GPT-4V 1st	Expert 2	0.77	0.73	0.78	0.51
Expert 2	Expert 1	0.96	0.97	0.96	0.92

Enlarged CDR was detected in 38% to 60% of photos by the expert graders across the datasets. Across all datasets, the experts identified enlarged CDR in 50% (Expert 1) and 39% (Expert 2) of images. Table 9 presents the accuracy and agreement of GPT-4V compared to expert graders in predicting the presence of an enlarged CDR using a binary yes/no classification. With respect to the ORIGA and RIM-ONE datasets, experts again achieved higher accuracy and agreement (kappa values ranging from 0.69–0.87) than GPT-4V (kappa values ranging from 0.27–0.65). In the ACRIMA dataset, however, GPT-4V performed more similarly to the experts in terms of agreement (GPT-4V vs. expert kappa values of 0.48 and 0.22, expert vs. expert kappa value of 0.35). For GPT-4V, enlarged CDR was detected in 45, 53, and 48 images for the ACRIMA, ORIGA, and RIM-ONE datasets, respectively. Notably, from these images, 44, 53, and 46 were detected as glaucoma, respectively. This indicates a strong correlation between GPT-4V glaucoma predictions and CDR classification, with most cases of enlarged CDR being classified as glaucoma across all 3 datasets (*P* values <0.001 for all datasets).

**Table 9:** Accuracy and Agreement of GPT-4V and Expert Graders for the Cup-to-Disc Ratio Assessment (Nonenlarged vs. Enlarged)

Model Prediction	Ground Truth	Accuracy	Sensitivity	Specificity	Cohen's Kappa
ACRIMA					
GPT-4V 1st	Expert 1	0.74	0.73	0.74	0.48
GPT-4V 1st	Expert 2	0.61	0.57	0.65	0.22
Expert 2	Expert 1	0.68	0.76	0.60	0.35
ORIGA					
GPT-4V 1st	Expert 1	0.68	0.68	0.68	0.35
GPT-4V 1st	Expert 2	0.64	0.60	0.67	0.27
Expert 2	Expert 1	0.85	0.97	0.76	0.69
RIM-ONE					
GPT-4V 1st	Expert 1	0.83	0.80	0.86	0.65
GPT-4V 1st	Expert 2	0.76	0.73	0.79	0.51
Expert 2	Expert 1	0.93	0.96	0.90	0.87

## Discussion

Our results suggest that GPT-4V has the potential to accurately detect glaucoma from fundus photographs and assess those photos for glaucoma-related features but with some limitations. GPT-4V accuracy in glaucoma detection was often lower than expert graders across all 3 datasets. Its agreement with itself (measured by comparing the 2 runs: *GPT-4V first* and *GPT-4V second*) and expert graders was also lower than the agreement between experts in 2 out of 3 of our datasets (ORIGA and RIM-ONE). It is important to note that both accuracy and agreement varied substantially across the datasets. This suggests that GPT-4V (as well as expert graders) are sensitive to the specific characteristics of each dataset, especially to the methods by which the glaucoma ground truth was determined.

One strength of the MM-LLMs like GPT-4V is their ability to generate a textual description of input images and a justification of model predictions, potentially providing an avenue for improved explainability of model decision-making. To help evaluate this ability, we asked GPT-4V’s to assess glaucoma-related fundus photo features and found that GPT-4V achieved high accuracy in distinguishing between gradable vs. ungradable images but more moderate accuracy in identifying rim thinning or enlarged CDR. The high accuracy of GPT-4V as compared with expert graders in determining image gradability may be due, in part, to the relatively low number of ungradable images (both experts graded 90%+ of the images as gradable). Indeed, looking at Cohen kappa suggests more moderate agreement between GPT-4V and the experts in gradability assessment. For identifying both rim thinning and enlarged CDR, GPT-4V vs. expert was comparable to expert vs. expert agreement in one of the datasets (ACRIMA) but lower than expert vs. expert agreement in the other 2 (ORIGA, RIM-ONE). Here again, the results suggest variability across datasets and a sensitivity to dataset characteristics and ground truth labels. It should also be noted, we observed that GPT-4V glaucoma predictions seemed highly related to CDR classification, if the CDR was considered enlarged, the eye was almost always classified as glaucomatous.

A potential avenue for improving GPT-4V accuracy and consistency across datasets is to perform additional fine-tuning on specific datasets of interest. Fine-tuning LLM models have previously been shown to improve performance in image-based and medical tasks.^33^, ^34^, ^35^, ^36^ Based on this previous work, additional fine-tuning and evaluation of general purpose LLMs (such as GPT-4V) on relevant datasets is critical to ensure their suitability for adoption in clinical settings. For many models, OpenAI and other vendors do provide tools and API to fine-tune existing models on additional datasets to improve their performance for specific tasks (e.g., glaucoma detection). Based on its current terms of service, OpenAI does state that users retain ownership of their data, data uploaded for fine-tuning will not be used for model training by OpenAI for its public models, and fine-tuned models are exclusive to data owners.^37^^,^^38^ Although these terms do address some issues related to data use, significant hurdles remain including, but not limited to, limited licenses and usage of existing datasets, privacy and security concerns, and compliance with relevant regulations.

It is also important to note that many LLM models, including GPT-4V, are designed to avoid offering medical predictions. Therefore, in our prompt, we explicitly state that our analysis is intended only for research evaluation and not for clinical use. Without this addition, the GPT-4V typically responded with disclaimer that it was not intended for medical use and refused to provide an analysis. Even when our prompt explicitly stated it was for research purposes, obtaining results GPT-4V often required multiple attempts. For our data, GPT-4V provided a glaucoma prediction and fundus assessment in 65% of cases on the first submission and required ≥2 submissions for the remaining cases. When it failed, the model might either provide generic information related to glaucoma and/or fundus evaluation not specific to the submitted image (17%) or simply refuse to perform the task (19%). Additionally, GPT-4V rarely provided precise probabilities for its glaucoma predictions, preventing us from performing a receiver operating characteristic analysis for its predictions.

Our study has several limitations. First, the predictions were made solely based on fundus images without incorporating any additional imaging, clinical, or demographic information. This approach may limit the diagnostic accuracy, because a comprehensive assessment for glaucoma typically requires multiple diagnostic modalities, and integrating additional data types, such as OCT and visual field testing could potentially enhance the model performance. Second, our analysis was conducted on a relatively small dataset. In future work, we aim to expand our analysis to include the entire public datasets, which will enable us to compare our results more comprehensively with other deep learning models evaluated on these datasets. Finally, our work serves as a snapshot of GPT-4V performance in glaucoma assessment at a single point in time. The GPT-4V predictions were collected over the course of a few weeks using the same GPT-4V release. Future software updates, algorithmic changes, and updated training data could lead to changes in how the model interprets and analyzes fundus images. As OpenAI continually refines GPT-4V to enhance its capabilities and address previously identified shortcomings, the diagnostic criteria and decision-making processes of the model may change or evolve. Given that GPT and other LLMs are being incorporated into health care systems, continuous monitoring and validation of AI models in medical applications is critical to ensure that updates lead to improved, consistent, and reliable performance across all intended use cases. In addition, the 3 public datasets used in our study (ACRIMA, ORIGA, and RIM-One v3) lack detailed demographic information about each image, limiting our ability to assess model performance across different populations. ACRIMA and RIM-One were collected from European cohorts, whereas ORIGA likely includes individuals of Chinese and Indian descent (Singapore). This limitation highlights the need for future studies with diverse demographic data to ensure the AI model's robustness and generalizability. In future work, we also aim to explore the potential of fine-tuning MM-LLMs like GPT-4V for clinical deployment. This includes addressing practical requirements, such as model interpretability, regulatory compliance, and real-world validation, to assess their potential for seamless integration into healthcare workflows.

Our findings indicate that GPT-4V has potential as a tool for glaucoma screening, detection, and clinical decision support through fundus image analysis. However, there are several limitations that may prevent adoption of the current GPT-4V model for fundus review in clinical settings. Refining the model through fine-tuning on glaucoma-specific datasets could further improve its accuracy in glaucoma detection, increasing its potential as a tool in ophthalmology.
