# Supplementary material for: Glaucoma Detection and Feature Identification via GPT-4V Fundus Image Analysis
Source: Ophthalmol Sci. 2024 Nov 29;5(2):100667. doi: 10.1016/j.xops.2024.100667 (PMC11773068; doi:10.1016/j.xops.2024.100667)
Supplement: Figure S9 [file mmc5.pdf]

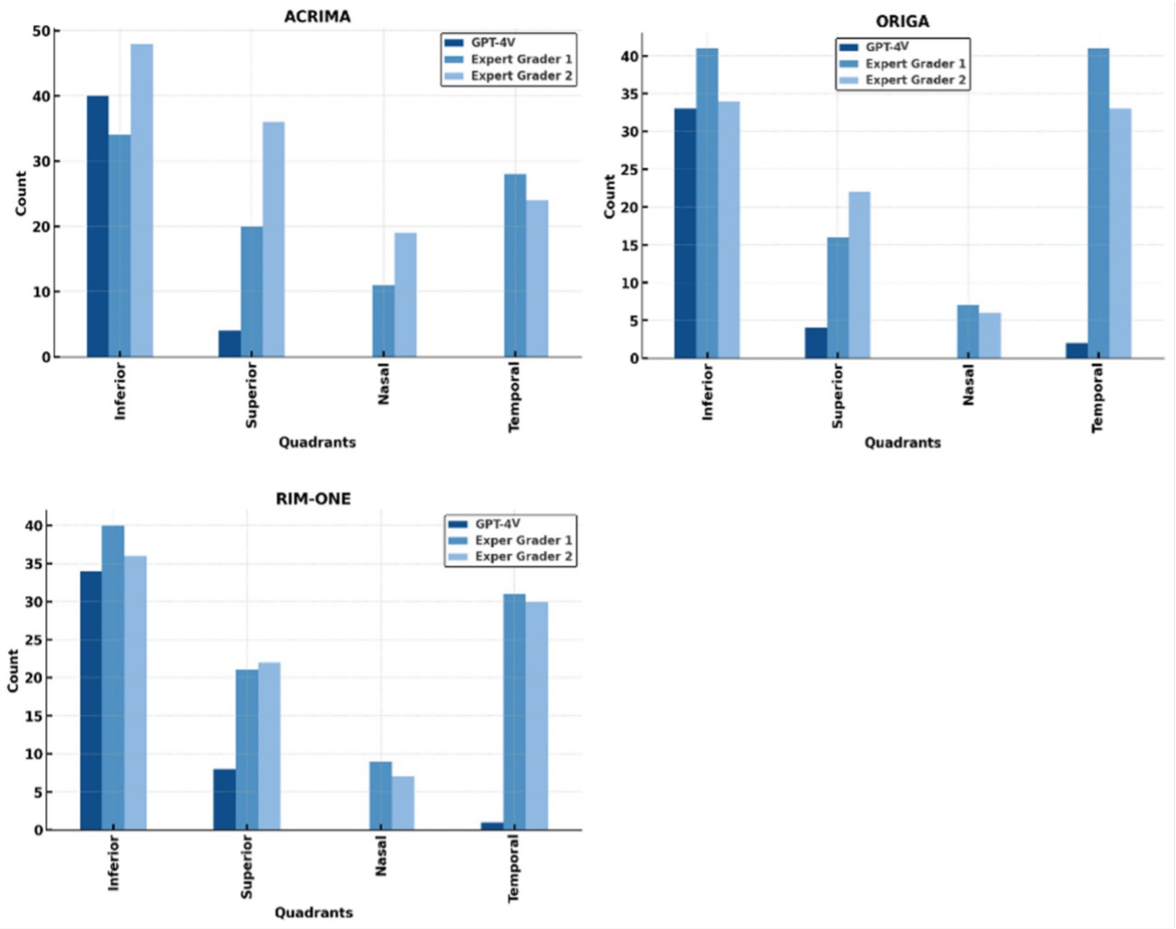

Figure S9: Comparison of Rim Thinning Detection by Quadrant Among GPT-4V and Expert Graders Across ACRIMA, ORIGA, and RIM-ONE Datasets
