# Supplementary material for: Glaucoma Detection and Feature Identification via GPT-4V Fundus Image Analysis
Source: Ophthalmol Sci. 2024 Nov 29;5(2):100667. doi: 10.1016/j.xops.2024.100667 (PMC11773068; doi:10.1016/j.xops.2024.100667)
Supplement: Table S6 [file mmc6.pdf]

Table S6: Comparison of Consistent and Inconsistent Glaucoma Predictions Between GPT-4V First and Second Evaluations

Consistent glaucoma prediction between GPT-4V 1st and GPT-4V 2nd

Image	Key Features		GPT-4V 1st	GPT-4V 2nd
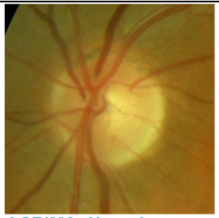 ACRIMA: Non-glaucoma<br>Experts: Non-glaucoma	Cup to Disc Ratio (CDR)	Nonenlarged /Enlarged	Enlarged	Enlarged
		Numerical Estimation	0.7	0.7
	Rim Thinning	Quadrants	Inferior	Inferior, Superior
		Clock hours	5 to 7	5 to 7 & 11 to 1
	PPA	Presence	No	No
		Location	N/A	N/A
	Glaucoma Prediction	Nonglaucoma/Glaucoma	Glaucoma	Glaucoma
		Probability of glaucoma	80	75

Image	Key Features		GPT-4V 1st	GPT-4V 2nd
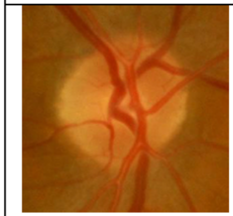 ACRIMA: Non-glaucoma<br>Experts: Non-glaucoma	Cup to Disc Ratio (CDR)	Nonenlarged /Enlarged	Nonenlarged	Nonenlarged
		Numerical Estimation	0.3 to 0.4	0.3 to 0.4
	Rim Thinning	Quadrants	No	No
		Clock hours	N/A	N/A
	PPA	Presence	No	No
		Location	N/A	N/A
	Glaucoma Prediction	Nonglaucoma/Glaucoma	Nonglaucoma	Nonglaucoma
		Probability of glaucoma	15-25	30

Inconsistent Glaucoma prediction between GPT-4V 1st and GPT-4V 2nd

Image	Key Features		GPT-4V 1st	GPT-4V 2nd
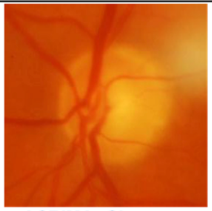 ACRIMA: Glaucoma<br>Experts: Glaucoma	Cup to Disc Ratio (CDR)	Nonenlarged /Enlarged	Nonenlarged	Enlarged
		Numerical Estimation	less than 0.5	somewhere above 0.5
	Rim Thinning	Quadrants	No	Yes: There could be some rim thinning
		Clock hours	N/A	-
	PPA	Presence	No	No
		Location	N/A	N/A
	Glaucoma Prediction	Nonglaucoma/Glaucoma	Nonglaucoma	Glaucoma
		Probability of glaucoma	30	60

Image	Key Features		GPT-4V 1st	GPT-4V 2nd
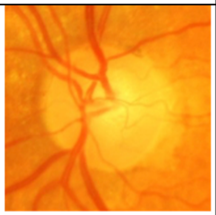 ACRIMA: Non-glaucoma<br>Expert 1: Glaucoma<br>Expert 2: Non-glaucoma	Cup to Disc Ratio (CDR)	Nonenlarged /Enlarged	Nonenlarged	Enlarged
		Numerical Estimation	0.3 to 0.4	0.6 to 0.7
	Rim Thinning	Quadrants	No	Inferior
		Clock hours	N/A	5-7
	PPA	Presence	No	No
		Location	N/A	N/A
	Glaucoma Prediction	Nonglaucoma/Glaucoma	Cannot determine	Glaucoma
		Probability of glaucoma	-	-
